# Scavenging on a pulsed resource: quality matters for corvids but density for mammals

**DOI:** 10.1186/s12898-017-0132-1

**Published:** 2017-06-15

**Authors:** Gjermund Gomo, Jenny Mattisson, Bjørn Roar Hagen, Pål Fossland Moa, Tomas Willebrand

**Affiliations:** 1 Nord University, 8049 Bodø, Norway; 2 Norwegian Institute for Nature Research (NINA), 7484 Trondheim, Norway; 3 Inland Norway University of Applied Sciences, 2418 Elverum, Norway

**Keywords:** Carrion ecology, Scavenging, Human subsidies, Pulsed resources, Gut piles, Scavenger community

## Abstract

**Background:**

Human food subsidies can provide predictable food sources in large quantities for wildlife species worldwide. In the boreal forest of Fennoscandia, gut piles from moose (*Alces alces*) harvest provide a potentially important food source for a range of opportunistically scavenging predators. Increased populations of predators can negatively affect threatened or important game species. As a response to this, restrictions on field dressing of moose are under consideration in parts of Norway. However, there is a lack of research to how this resource is utilized. In this study, we used camera-trap data from 50 gut piles during 1043 monitoring days. We estimated depletion of gut piles separately for parts with high and low energy content, and used these results to scale up gut pile density in the study area. We identified scavenger species and analyzed the influences of gut pile quality and density on scavenging behavior of mammals and corvids (family Corvidae).

**Results:**

Main scavengers were corvids and red fox (*Vulpes vulpes*). Parts with high energy content were rapidly consumed, mainly by corvids that were present at all gut piles shortly after the remains were left at the kill site. Corvid presence declined with days since harvest, reflecting reduction in gut pile quality over time independent of gut pile density. Mammals arrived 7–8 days later at the gut piles than corvids, and their presence depended only on gut pile density with a peak at intermediate densities. The decline at high gut pile densities suggest a saturation effect, which could explain accumulation of gut pile parts with low energy content.

**Conclusions:**

This study shows that remains from moose harvest can potentially be an important food resource for scavengers, as it was utilized to a high degree by many species. This study gives novel insight into how energy content and density of resources affect scavenging patterns among functional groups of scavengers.

## Background

Human subsidies such as food waste, crop residuals, feeding stations for game species and carcass remains from hunting, are abundant in large quantities worldwide. Predictable Anthropogenic Food Subsidies (PAFS) are resources offered intentionally, or unintentionally, to wild animals by humans and are predictable in time and/or space [1]. PAFS can increase body condition, fecundity and survival of scavengers [2–7], and in the end lead to multiple changes of processes in the ecosystem. For example, predator species can increase in abundance through buffered temporal variability in food [8, 9], leading to altered predator–prey dynamics potentially affecting whole communities [1]. PAFS could also be negative for certain species when artificially increased populations disrupts the social system [10].

In temperate ecosystem, scavenging on remains from ungulate harvest are potentially important as PAFS [11–14]. A regulated harvest of ungulates and historical low numbers of large carnivores in Fennoscandia have resulted in large populations of ungulates, especially the moose (*Alces alces*) have shown an unprecedented increase the last 75 years [15, 16]. About 35,000 moose are annually harvested during a few weeks in September and October in Norway. Internal organs including lungs, intestines, liver and sometimes the heart are removed at the kill site and these gut piles dominate the amount of available moose carrion in autumn [17, 18]. Gut piles from moose are a predictable and high amplitude temporal resource pulse during a time when low temperatures reduce the proliferation of insects, bacteria and fungi on the remains [18–20]. Autumn is also a critical period for many carnivores and scavengers, especially for young individuals. Autumn mortality in juvenile corvids is high, [21], and starvation is an important mortality factor in juvenile raptors as goshawks (*Accipiter gentilis*) [22] and golden eagles (*Aquila chrysaetos*) [23]. Mortality in dispersing juvenile American martens (*Martes americana*) was related to body condition [24], and low food availability increase trapping vulnerability of the European pine marten (*Martes martes*) [25]. Hence, gut piles might increase juvenile survival of scavengers by increasing availability of food resources. In addition, adaptations by scavengers, e.g. storing of body fat or food caching may lead to prolonged effects of food pulses [26–30].

Gut piles from harvested moose at kill sites have recently become a conservation concern, and this artificial support of scavenging species can have negative impact on other species. For example, ground nesting birds may suffer increased nest predation as a consequence of increased densities of scavenging generalist predators [31–33]. In Scandinavia, the red fox (*Vulpes vulpes*) is of special concern, as it has been documented to reduce breeding success through nest and chick predation [32, 34] and as a threat to the arctic fox (*Vulpes lagopus*) through interference and resource competition [35]. An increasing number of landowners now enforces restrictions on field dressing of ungulates, but the potential effect of this management policy is unclear.

The aim of this study was to investigate scavenging patterns on the large quantities of gut piles from the moose harvest in Norway. We hypothesize that a wide range of facultative scavengers will use this human created resource, and that avian species will be the first to detect the remains. Firstly, we measured how the different species in the scavenger community utilize the resource by comparing the arrival time and group size of different species at gut piles. Secondly, we quantified the rate of gut pile depletion and the temporal change in gut pile density as an indication on the potential effects pulsed resource can have on the scavenging community [36, 37]. Thirdly, gut piles contains different tissues as fat, muscle and connective with varied energy content, which could be expected to influence foraging behavior [19, 38–41]. Therefore we evaluated how temporal variation in gut pile density and energy content influenced the foraging patterns of different functional groups of scavengers.

## Methods

### Study area

The study was conducted in an area of 65 km^2^ 90–485 m.a.sl. within the Ogndal valley in central Norway (63.95 N–64.03 N, 11.76 E–11.97 E). At elevations below 165 m the geology is dominated by marine deposits, mostly agriculture land interspersed with commercially managed forest, mainly Norway spruce (*Picea abies*). The area above the marine deposits is dominated by coniferous forest (Norway spruce and Scots pine *Pinus sylvestris*) interspersed with bogs. Potential scavenging species are red fox, European badger (*Meles meles*), pine marten, golden eagle, white-tailed eagle (*Haliaeetus albicilla*), northern goshawk and corvid species. There is no obligate scavengers in Scandinavia. Ungulate present includes moose, roe deer (*Capreolus capreolus*), and occasional red deer (*Cervus elaphus*). Free ranging semi-domesticated reindeer (*Rangifer tarandus*) are usually present in the area from October to May. Eurasian lynx (*Lynx lynx*) populations were relatively low during the study period [42] while wolverine (*Gulo gulo*) and brown bear (*Ursos arctos*) were only sporadically registered within the area. Average monthly temperature varied between 3–5, 1–3 and −5 to 0 °C in October, November and December, respectively. Monthly precipitation ranged from 30 to 148 mm. Snow covered the ground periodically each winter and snow layer >25 cm was restricted to a few days.

The number of moose harvested in the study area was 61 in 2012, 62 in 2013 and 60 in 2014, resulting a pooled average of 0.94 moose/km^2^. Calves constituted 60% of the harvest. The hunting season was closed during 1 week at the peak rut to avoid disturbance. The first hunting period was starting at September 25th, and the second hunting period began October 10 and lasted to October 30 in 2012 and to November 14 in 2013 and 2014. Head, legs, hide and often the heart were brought out with the carcass, while gut piles, containing stomachs and intestines, including visceral fat, and other internal organs were usually left in the field. The lowest energy density of the gut piles is in the stomach tissue with ~0.5 kcal/g, about half of what is found in muscle and liver. Lungs have an intermediate energy density of ~0.85 kcal/g [43]. Highest energy density is found in fat (~9 kcal/g dry weight), however the energy density of fat tissue is lower, dependent on water content [44]. Estimated biomass of moose gut piles in our study area (rumen contents excluded; calculated as Wikenros et al. [18]) was higher (33 kg/km^2^) compared to the surrounding region (20 kg/km^2^ in the county of Nord-Trøndelag [17]).

### Scavenging observations

**Fig. 1:**
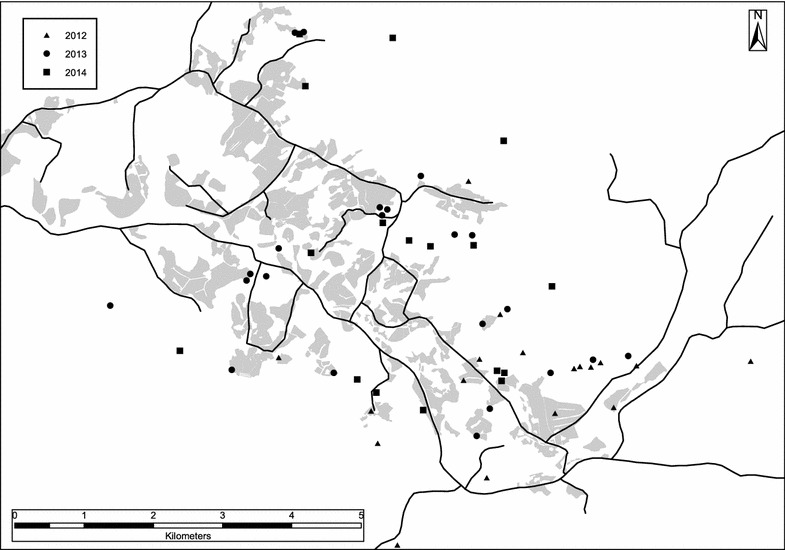
Location of gut piles with camera-traps 2012–2014. Map showing the location of moose gut piles with camera-traps, N = 50 out of totally 183 moose killed within the study area. Locations of moose gut piles without camera-traps were not recorded. *Grey* show agricultural areas. *Lines* are roads

Camera traps (Reconyx Hyperfire PC 900 in 2012 and Wingcam II TL in 2013/2014) were set up on 50 gut piles, totaling 1043 monitoring days, during the hunting season in 2012–2014 (Fig. 1). Cameras were set up by the hunters before they left the dressing site (1 camera per site) and were placed 4–6 m away from the gut pile and 1−1.5 m above ground. The cameras were programmed to take a picture every 10 min and also when triggered by the motion sensor, with a 2 min delay between triggers to maintain battery and memory card capacity. Cameras were removed when only the rumen contents remained and occasionally smaller pieces of the intestines. All pictures were examined and the number of individuals present of each species in each picture was registered. To estimate daily energetic quality of the remains of the gut pile, we visually evaluated the first picture of each day. Gut piles were then categorized into two classes: (1) high energy content (parts with high energy density like fat, liver and lungs still present), (2) low energy content (only stomach and/or intestines present, including rumen content).

### Gut pile depletion curves and gut pile density

Kaplan–Meier curves were used to estimate gut pile depletion for class 1 and 2 separately due to different usage by different scavengers. Right censored events occurred when presence of high energy parts were uncertain due to snow cover or when the gut pile was moved so that only low energy parts were visible on pictures, this was included as a right-censored event in the Kaplan–Meier analyses for class 1. Class 2 was categorized as depleted when only rumen content was left.

To evaluate if depletion varied between years or hunting periods, we compared the depletion rates by using Cox proportional hazard models. We tested for the difference in depletion rate between class 1 and 2 in different years. There were no significant differences between years for either class (coxph class 1; year 2012 v 2013: z = 1.083, p = 0.279, year 2012 v 2014: z = −0.843, p = 0.399, coxph class 2; year 2012 v 2013: z = −0.043, p = 0.966, year 2012 v 2014: z = −0.819, p = 0.413), and data were pooled for all years. We further compared depletion rates between the early (Sep 25–Oct 1) and late (Oct 10–Nov 14) hunting period. All analyses were done in R [45] with the package survival [46]. The function cox.zph was used to evaluate the assumptions of constant proportional hazard models.

Secondly, we calculated gut pile density throughout the hunting season (all years pooled). Separate estimates were calculated for the parts with high (class 1) and low (class 2) energy content. The change in density of the two classes was calculated by reducing the accumulated gut piles from harvest with the Kaplan–Meier depletion estimates for each day of the hunting season.

### Scavenging patterns

For each scavenging species, we estimated the proportion of gut piles visited, the mean number of days with visits and the maximum number of individuals recorded at one time for each day. Maximum number of individuals was used to investigate variation in aggregation of individuals between species.

To analyze if daily presence of scavengers responded primarily to days since the moose was killed or to density of gut piles we used binomial generalised linear mixed-effects models (GLMM; in R-package lme4 [47]). We analysed the daily probability (N = 1043) for each scavenging species to visit gut piles (0 or 1, where 1 is defined as ≥1 pictures including the species). We pooled scavenging species into three functional groups: (1) mammals, (2) large corvids [magpie (*Pica pica*), hooded crow (*Corvus cornix*) and common raven (*Corvus corax*)] and (3) small corvids [Eurasian jay (*Garrulus glandarius*) and Siberian jay (*Perisoreus infaustus*)] for separate analyses. Raptors were not included in this analysis due to small sample sizes. We included gut pile ID (N = 50), nested under year (N = 3), as random intercept in the model to account for possible variation in scavenger densities between the years of the study and for repeated measures at the same gut pile within a single year. As gut pile densities were not independent of days since harvest we did not combine the two variables in the same model but rather viewed them as competing models, evaluated by AIC_c_ values. The effect of age and density was evaluated through a second order polynomial, and AIC was used to determine if the non-linear (second order) was better than the linear (first order). Gut piles were removed from the analyses when only rumen content remained.

## Results

**Fig. 2:**
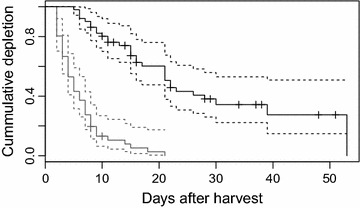
Depletion of gut pile parts with high and low energy content. *Grey* high energy content, *black* low energy content. *Dashed lines* show 95% confidence intervals

The gut pile parts with high energy content (class 1) were depleted at significantly higher rate compared to parts with low energy content (class 2) (coxph: z = −7.504, p < 0.001). Already 10 days after the moose were killed, only 15% of the gut piles contained parts with high energy content, although 90% still had remains. Ten percent were depleted (i.e. only rumen content left) (Fig. 2). There was no significant difference (coxph: z = 1.333, p = 0.183) in depletion of the class 2 parts between the hunting periods. Depletion of the class 1 part tended to go faster in the second hunting period, but differences could not be estimated because coxph model assumptions were violated.

**Fig. 3:**
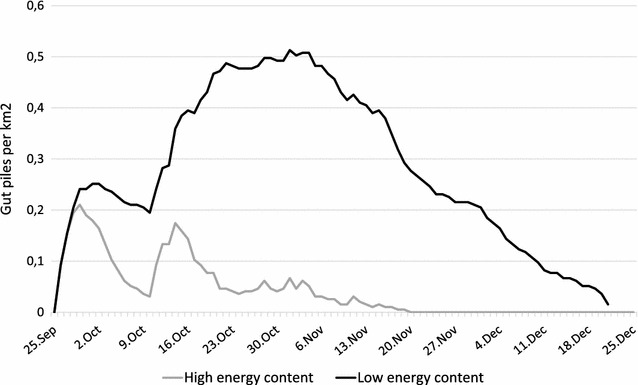
Temporal development of the availability of gut pile parts with high and low energy content. Years are pooled

Density of gut piles with high energy content parts remaining was highest during the first hunting period, whereas low energy parts lasted longer and at higher density (Fig. 3).

### Scavenging patterns

In total, 15 species scavenged on the gut piles. Magpie, Eurasian jay, hooded crow and common raven were the most common avian scavengers while red fox was the most common mammal (Table 1). In addition to species in Table 1, arctic fox, American mink (*Neovison vison*) and domestic dog (*Canis lupus familiarizes*) were registered scavenging on one gut pile each. Pine martens only visited a quarter of the available gut piles but were often observed many days to the same gut pile (Table 1). The same pattern (high species revisiting rate) was also observed for several corvids species. Crows, and partly ravens and magpie, were the only species aggregating in groups, up to 27, 16 and 12 individuals were recorded at one time respectively. Jays, raptors and mammals were usually only present one individual at a time (Table 1). Interestingly, even domestic cats (*Felis catus*) visited some of the gut piles, and returned frequently to the same gut pile.

**Table 1:** Species documented on gut piles (N = 50) from moose harvest in Central Norway in 2012–2014

Species^a^	Latin name	% visited	Days present	Max no. ind.
Birds				
Magpie	*Pica pica*	100	10.9 (7.1)	3 (1–12)
Eurasian jay	*Garrulus glandarius*	90 (82–96)	7.6 (7.9)	1 (1–5)
Hooded crow	*Corvus cornix*	90 (82–94)	5.2 (3.2)	8 (1–27)
Raven	*Corvus corax*	70 (55–81)	2.8 (2.1)	3 (1–16)
Siberian jay	*Perisoreus infaustus*	10 (6–18)	2.6 (0.9)	1 (1–2)
White-tailed eagle	*Haliaeetus albicilla*	20 (17–27)	2.6 (2.3)	1 (1–2)
Golden eagle	*Aquila chrysaetos*	16 (9–27)	1.4 (0.5)	1 (1–2)
Goshawk	*Accipiter gentilis*	6 (0–19)	2.3 (1.3)	1
Mammals				
Red fox	*Vulpes vulpes*	68 (45–78)	4.0 (2.6)	1 (1–3)
Badger	*Meles meles*	40 (35–55)	4.4 (3.5)	1 (1–2)
Pine marten	*Martes martes*	24 (6–35)	7.5 (4.9)	1 (1–2)
Domestic cat	*Felis catus*	6 (0–13)	9.0 (4.6)	1

Proportion of gut piles visited (% visited) is presented with all years pooled and range for the different years, while numbers of days with visits per gut pile (days present) is presented as mean (±SD) and daily maximum number of individuals recorded at one time (Max no. ind) as the median and range (min–max)

^a^Arctic fox (*Vulpes lagopus*), American mink (*Nivea vision*) and domestic dog (*Canis lupus familiaris*) were registered scavenging on one gut pile each

**Fig. 4:**
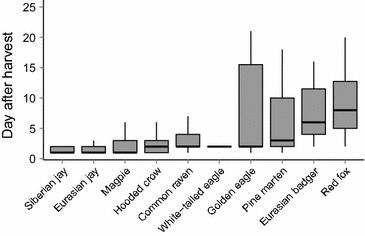
Arrival time at gut piles from moose harvest (days after harvest) by different scavenger species. Based on 50 harvested moose in central Norway in 2012–2014. Box plots show median (*bold horizontal lines*), interquartile range (*box*), and range up to 1.5 times interquartile range (*bars*)

**Fig. 5:**
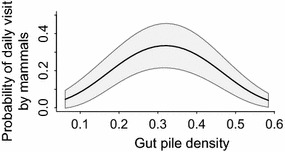
Probability of scavenging mammals to visit gut piles in relation to gut pile density. Gut piles remaining after field dressing of hunted moose in Central Norway in 2012–2014

Scavenging birds (raptors, large corvids and small corvids) arrived 5–8 days earlier at gut piles than mammals did (ANOVA: F_3,258_ = 30.5, p < 0.001), while there was no difference in arrival time between groups of birds (eagle sp.; TukeysHSD: p = 0.08–0.4; Fig. 4). The probability of mammals to visit gut piles was best explained by density of gut piles, showing a strong non-linear response peaking at around median densities (Fig. 5; β1 = −3.3, SE = 3.7; β2 = −19.0, SE = 3.8). Gut pile age had no effect on daily visits by mammals (ΔAIC_c_ = 29.9, c.f. Null model: ΔAIC_c_ = 29.5). There were some variance in random intercept between gut piles (1.4, 1.2 SD) and a tendency for variation between years (0.06, 0.24 SD).

**Fig. 6:**
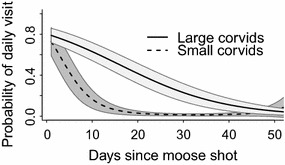
Probability of scavenging corvids to visit gut piles in relation to gut pile age. Gut piles remaining after field dressing of hunted moose in Central Norway in 2012–2014

In contrast, the probability of both small and large corvids to visit gut piles was only influenced by days since harvest. Model with gut pile density increased AIC_c_ by 172 and 108 for small and large corvids respectively. The daily presence of small corvids decreased rapidly (Fig. 6; β1 = −46, SE = 4.2; β2 = 29, SE = 3.1), while the daily presence of large corvids decreased in a linear manner and more gradually with increasing age of the gut pile (non-linear ΔAIC_c_ = 1.4, β = −0.09, SE = 0.009).

The general probability of visits varied between gut piles (4.3, SD 2.1 for small corvids and 1.3, 1.2 SD for large) but there was no variance between years thus this variable was removed from the analyses.

## Discussion

We have shown that gut piles from moose hunt are rapidly detected, and parts with high energy content are removed in less than 3 weeks. Gut piles constitute a large amount of food and are likely to have a positive influence on several of the scavenger populations. This pulse of food increased resource availability for scavenging species during a 3-month period. It seems safe to conclude that moose gut piles fit the PAFS concept.

In this study, corvids, red fox, pine marten and badger were the main scavenger species. Northern ecosystems lack obligate scavengers and the low abundances of large scavenging carnivores opens up for smaller carnivores [13, 18, 48, 49]. The high moose harvest in Scandinavia is one, if not the most, important food resource for scavengers, making it unique compared to boreal areas in other parts of the world. There has been high moose harvest for about 40 years, and the harvest periods do not change much between years. We believe that the scavenging species in this ecosystem have adapted to this resource, and it may be an important food buffer potentially reducing juvenile mortality [21–25, 50]. The high energy content of the visceral fat in the gut piles is easily converted to body fat storage in mammals, reducing the risk of starvation during winter bottle-necks [30]. Alternatively, the remains can be cached and utilized later [26–29, 51–53].

In our study, gut pile parts with high energy content were utilized rapidly, primarily by corvids. Corvids were present at all gut piles shortly after harvest, and had access to all parts of the gut pile. Corvid presence declined with time probably reflecting reduction in gut pile quality. Optimal foraging theory predicts that only the energy rich parts should be utilized before moving to a new patch if available, as observed in both predators and scavengers [38, 40, 41]. Our results suggest that corvids move to a more recent harvest site nearby rather than staying and feeding on remains of poorer quality. Stomachs and intestines have high content of connective tissue, and corvids might have limited ability to digest collagen compared to mammal scavengers [54]. Dependent on size, corvid species might also be restricted by limited beak strength [55]. This also reflects the benefits of direct access to harvest remains for birds compared to whole carrions where access to parts with high energy content may require opening by larger species [56].

The late arrival of mammals seems to limit their access to parts with high energy content, already consumed by avian scavengers. Mammals responded primarily to gut pile density and daily presence peaked at intermediate densities. Reduced mammal presence at high gut pile densities might indicate a saturation effect, comparable to seed predation during masting events [57]. On the other hand, accumulation of gut pile parts with low energy content lead to longer resource pulse duration, with possible prolonged positive effects on mammal scavengers [37]. Hence, even a reduction in gut pile density may reduce the potential positive effects on the density of mammalian scavengers. Nonetheless, corvids probably have an important ecological impact in limiting gut pile availability to other scavengers through resource competition [58]. Hence, lower corvid abundance may benefit mammalian scavengers. Large scavenging predators (wolverine, brown bear) were only present sporadically in our study area and were not observed at any of the gut piles. This contrasts to comparable studies on autumn pulses of gut piles, where bear species were the main mammal scavengers [12, 59]. Red fox is the main mammal scavenger in other studies from northern Europe [18, 19, 48], and in studies of human provided subsidies in general [60].

Direct negative impacts of scavenging on gut piles from harvested moose is potential lead poisoning arising from bullet remains [59, 61]. An estimated deposit of 182 kg of lead in moose gut piles yearly in Scandinavia could be a management concern regarding scavenging species [62]. For example, golden eagles show an increase in blood lead levels during the moose hunting season, and might experience increased mortality both through lethal and sub lethal doses of lead [63]. Legislation banning the use of lead ammunition or forcing removal of gut piles would effectively reduce the risk of lead poisoning. However, the latter would in addition result in a radical reduction regarding food abundance for scavenging species, possibly with larger ecosystem impact [64, 65]. In addition, anthropogenic resources can alter wildlife–pathogen dynamics and create opportunities for cross-species transmission of pathogens [66]. The protozoan parasites *Toxoplasmoso gondii*, *Giardia* spp. and *Cryptosporidium* spp. found in moose and other cervids [67, 68] can infect several scavenger species [69–71]. On the other hand, red fox use of anthropogenic food sources may indirectly reduce the prevalence of the zoonotic tapeworm *Echinococcus multilocularis* in foxes if it results in reduced predation on small rodents, the intermediate hosts of the parasite [72, 73]. Interestingly, prevalence of *E. multilocularis* in small rodents increases during autumn and winter [74], the period gut piles are present. However, possible effects of gut piles on wildlife–pathogen dynamics are probably diverse [66], but should be considered.

## Conclusions

In this study, we show that gut piles left at the kill site after moose harvest are an intensively used food source by a range of scavenging birds and mammals during autumn and early winter. This study also provides novel insight into how quality and density of carrion affect scavenging patterns among functional groups of scavengers. Enforcing a removal of gut piles by hunters will reduce food supply in a critical period for several scavenging species. This may have direct negatively effects on several scavenging species utilizing this predictable food resource, but it is difficult to predict the long-term effect on the relative abundance of scavengers. It could potentially reduce less wanted species as corvids and red fox, but also impact eagles and wolverines. Reducing the amount of gut piles in the landscape would reduce the potential risk of lead poisoning [59, 61], but could also be mitigated by using lead free bullets. However, to what extend this pulsed resource is important for the overall survival, reproduction and population dynamics of both scavenging and potential prey species needs further investigation. Ignoring these PAFS would make the understanding of the food web structure and dynamics in the boreal forest difficult.
